# Plasma S100β and neuron-specific enolase, but not neuroglobin, are associated with early cognitive dysfunction after total arch replacement surgery: A pilot study

**DOI:** 10.1097/MD.0000000000025446

**Published:** 2021-04-16

**Authors:** Zilin Wan, Yaxiong Li, Huishun Ye, Yunfeng Zi, Guojing Zhang, Xiaoyan Wang

**Affiliations:** a Department of Cardiovascular Anesthesiology; b Department of Cardiovascular Surgery, Yan’An Hospital, Kunming Medical University, Kunming, 650051, Yunnan, China.

**Keywords:** neuroglobin, neuron-specific enolase, postoperative cognitive dysfunction, predictive value, S100β protein, sensitivity and specificity, total arch replacement

## Abstract

To investigate whether plasma concentrations of S100β protein, neuron-specific enolase (NSE), and neuroglobin (NGB) correlate with early postoperative cognitive dysfunction (POCD) in patients undergoing total arch replacement.

This prospective study analyzed 40 patients who underwent total arch replacement combined with stented elephant trunk implantation at our hospital between March 2017 and January 2019. Cognitive function was assessed using the Mini-mental State Examination (MMSE) preoperatively, on the day after extubation and on day 7 after surgery. Plasma levels of S100β, NSE, and NGB POCD were assayed preoperatively and at 1, 6, and 24 hours after cardiopulmonary bypass. POCD was defined as a decrease of at least 1 unit in the MMSE score from before surgery until day 7, and patients were stratified into those who experienced POCD or not. The 2 groups were compared in clinicodemographic characteristics and plasma levels of the 3 proteins.

Plasma levels of all 3 biomarkers increased significantly during and after cardiopulmonary bypass. Levels of S100β and NSE, but not NGB, were significantly higher in the 15 patients who showed POCD than in the remainder who did not. For prediction of early POCD, S100β showed an area under the receiver operating characteristic curve (AUC) of 0.71 (95% confidence interval [CI] 0.55–0.87), sensitivity of 48%, and specificity of 87%. The corresponding values for NSE were 0.77 (95%CI 0.60–0.94), 92%, and 67%. Together, S100β and NSE showed an AUC of 0.81 (95%CI 0.66–0.96), sensitivity of 73%, and specificity of 80%. NGB did not significantly predict early POCD (AUC 0.62, 95%CI 0.43–0.80).

Plasma S100β protein and NSE, but not NGB, may help predict early POCD after total arch replacement.

## 1 Introduction

Postoperative cognitive dysfunction (POCD) is one of the most frequent complications after total arch replacement combined with stented elephant trunk implantation while the patient is under deep hypothermia circulatory arrest. POCD may occur in approximately 37% of patients undergoing aortic arch surgery, and 12% of all patients suffer permanent neurological disability after this procedure.^[1]^ Identifying biomarkers linked to POCD may help clinicians stratify patients by risk and intervene quickly. Such biomarkers may also provide clues to help elucidate the mechanisms behind POCD, leading to prophylactics or treatments.

One such biomarker may be the plasma level of S100 protein, a dimer of α and/or β subunits that was first discovered in bovine brain in 1965.^[2]^ The ββ dimer (S100β) is found mainly in glial and Schwann cells, where it is involved in the synthesis of fibrin and intermediate filaments^[3]^ as well as calcium-mediated signaling.^[4]^ Damage to the blood–brain barrier causes its release from cells into the cerebrospinal fluid or blood,^[5,6]^ and its concentration levels positively correlate with POCD after cardiac surgery.^[7]^

Another potential POCD marker is neuron-specific enolase (NSE), which participates in glycolysis in neuroendocrine cells and cells of the central nervous system. Under normal conditions, its plasma levels are extremely low; when neurons are damaged, its levels in cerebrospinal fluid and blood increase,^[8,9]^ and such increases are associated with POCD.^[10]^

A third potential POCD biomarker is neuroglobin (NGB), discovered in 2000 as a novel globin that delivers oxygen in the nervous system.^[11,12]^ It is highly expressed in the frontal lobe, hypothalamus nucleus, and thalamus,^[13]^ and its serum concentration increases after nerve cell damage.^[14]^

Here we examined whether the levels of these potential biomarkers may help predict early POCD in patients who have undergone total arch replacement.

## 2 Methods

### 2.1 Patients

This prospective study considered patients older than 18 years who underwent total arch replacement combined with stented elephant trunk implantation under cardiopulmonary bypass (CPB) and deep hypothermia circulatory arrest (DHCA) between 20 March 2017 and 14 January 2019 at our hospital. Patients were excluded if they had

(1) a history of stroke in the prior 6 months or any other central nervous system disease, including Parkinson disease, Alzheimer's disease, and encephalitis;
(2) a psychotic disorder or family history of psychotic disorder; or
(3) preoperative cognitive impairment,^[15]^ defined as a Mini-mental State Examination^[16]^ (MMSE) score < 17 if the individual was illiterate, <20 if the individual had only primary school education, or <24 if the individual had at least a middle school education.

Enrolled patients were exited from the study if they died within 24 hours after surgery or suffered stroke after surgery.

This cohort study was approved by the institutional ethics committee of Yan’An Hospital affiliated to Kunming Medical University (2018-069-01). At admission, participants received a written and oral explanation of the study and gave written informed consent to be enrolled in the study.

### 2.2 Anesthesia, surgery, and analgesia after surgery

In the operating room, patients were routinely monitored in terms of electrocardiography, pulse oxygen saturation, cerebral oxygen saturation, central venous pressure, and invasive arterial blood pressure in the left radial artery as well as the left or right femoral artery. Anesthesia was induced by intravenous injection of 0.1 mg/kg midazolam, 0.3 mg/kg etomidate, and 0.7 to 1.0 μg/kg sufentanil, and muscles were relaxed using 0.6 mg/kg rocuronium. After intubation, mechanical ventilation was performed at a tidal volume of 8 to 10 mL/kg and frequency of 10 to 12 times per minute to maintain the end expiratory pressure of carbon dioxide at 30 to 35 mm Hg. Anesthesia was maintained using the following drugs and doses (per kg per hour): cisatracurium, 0.1 to 0.15 mg; sufentanil, 0.7 to 1.0 μg; propofol, 2.0 to 4.0 mg; and dexmedetomidine, 1.0 μg.

Median sternotomy was performed, then heparin (3 mg per kg) was injected intravenously. CPB was established by exposing the axillary artery, then anastomosing it with a graft for artery perfusion. Next, a double-staged cannula (32/42Fr, Medtronic, Minneapolis) was inserted into the right auricular appendage for venous drainage. The bypass circuit was completed with a roll pump (S5, Stockert, Munich, Germany) and membrane oxygenator (BB841, Medtronic). The circuit was primed with Ringer's solution containing sodium acetate, hydroxyethyl starch, and albumin.

The aorta was cross-clamped at a nasopharyngeal temperature of 34°C, and Del Nido cardioplegia was achieved by perfusing the left and right coronary arteries with priming fluid (20 mL per kg). Selective antegrade cerebral perfusion was performed at 5 to 12 mL per minute per kg via the innominate artery when the nasopharyngeal temperature was 25°C and the anal temperature was below 28°C. Total arch replacement combined with stented elephant trunk implantation was performed. During circulatory arrest, left radial artery pressure was maintained at 20 to 30 mm Hg. Perfusion of the lower body was resumed after anastomosis of the graft and descending aorta.

The left common carotid artery was anastomosed with the graft. When mixed venous oxygen saturation exceeded 65%, rewarming began. Then the ascending aorta was anastomosed using an artificial four-branch blood vessel. The aorta cross-clamp was removed. Vasoactive drugs were used as necessary to stabilize circulation.

Sufentanil was administrated after surgery. Sufentanil was stopped before extubation in order to keep the patients conscious.

### 2.3 Assessment of neurological function

POCD was diagnosed based on MMSE score. The MMSE tests abilities of orientation, memory, attention and calculation, memory, and language. Patients completed the MMSE before surgery, at 1 hour after extubation and on day 7 after surgery.

The highest possible MMSE score is 30. A patient was considered to have early POCD if either postoperative MMSE score was lower than the preoperative score by at least 1 unit.^[17]^

All MMSE evaluations were conducted by the same investigator (HSY), who was specially trained by an experienced neurologist before the study began.

### 2.4 Blood analyses

Blood (3 mL) was sampled via central vein catheter after induction of anesthesia, immediately after rewarming to 36°C, and at 1, 6, and 24 hours after CPB. Samples were centrifuged at 4000 rpm for 10 minutes, then plasma was transferred to an Eppendorf tube and stored at −80°C. Thawed samples were assayed using commercial ELISA kits for S100β (catalog no. KE00103, Sanying Proteintech Biological Engineering, Wuhan, China), NSE (KE00050, Sanying Proteintech), and NGB (SEA606, Hu Cloud-Clone, Wuhan, China).

### 2.5 Statistical analysis

Data were analyzed using SPSS 17.0 (IBM, Chicago, IL) and GraphPad Prism 6.0 (GraphPad Software, San Diego). Continuous data were expressed as mean ± SD. Intra-group differences between time points were assessed for significance using repeated-measures ANOVA, while inter-group differences were assessed using an unpaired *t* test. Categorical data were compared between groups using the chi-squared test. Differences associated with *P* < .05 were considered significant.

Univariate and multivariate forward logistic regression was used to identify potential risk factors of POCD. Variables associated with *P* < .05 in univariate analysis were included in the multivariate model. Where appropriate, results were reported as odds ratios (ORs) with 95% confidence intervals (CIs).

Receiver operating characteristic curves were generated to assess the ability of peak levels of biomarkers to predict early POCD. Peak values were defined as the highest values during or after CPB. The 2 markers showing the highest sensitivity and specificity on their own were then also tested in combination.

## 3 Results

**Figure 1:**
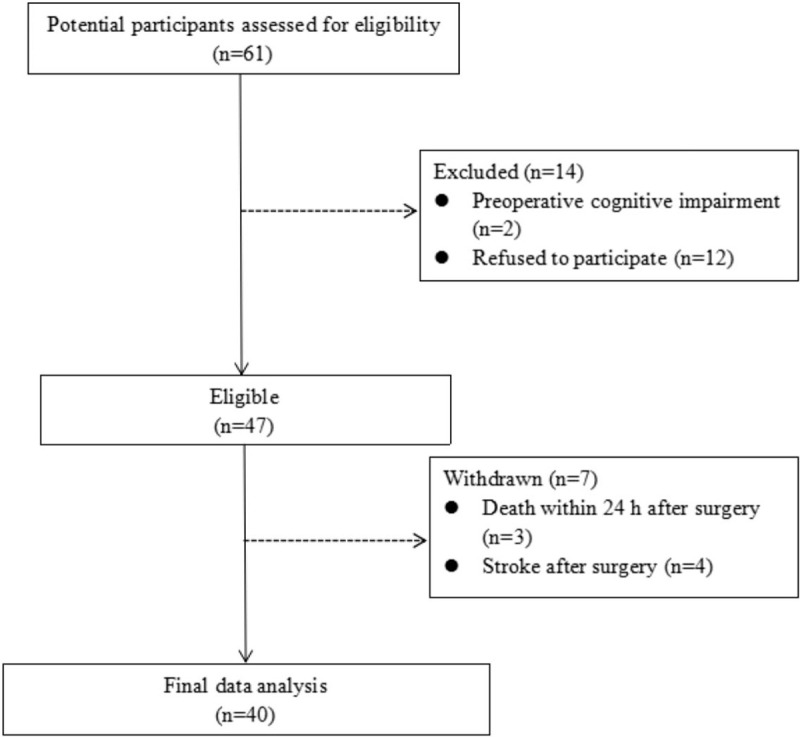
Flow diagram of patient screening and enrollment.

Of the 61 patients who underwent total arch replacement combined with stented elephant trunk implantation during the recruitment period, 14 were excluded because 2 had preoperative cognitive impairment and 12 refused to participate in the study. After excluding another 3 patients because they died within 24 hours after surgery and another 4 because they suffered stroke after surgery, we included 40 patients (31 men) in the final analysis, including 34 (85.0%) with a history of hypertension and 27 (67.5%) who experienced respiratory failure before surgery (Table 1). These patients had a mean age of 51 years (Fig. 1), and POCD occurred in 15 (37.5%).

**Table 1:** Characteristics of patients and surgical procedures.

Characteristic	Total (n = 40)	POCD (n = 15)	Non-POCD (N = 25)	*P*
Demographics				
Age, yr	51 ± 11	50 ± 10	52 ± 12	.58
Male	31 (77.5)	9 (60.0)	22 (88.0)	.04
Education level				
Illiterate	4 (10.00)	1 (6.67)	3 (12.00)	.59
Primary school	14 (35.00)	5 (33.33)	9 (36.00)	.86
Middle school or higher	22 (55.00)	9 (60.00)	13 (52.00)	.62
Body mass index, kg/m^2^	25.16 ± 4.60	25.79 ± 6.26	24.78 ± 3.33	.51
Current smoker	7 (17.50)	1 (6.70)	6 (24.00)	.16
Current drinker	4 (10.00)	2 (13.33)	2 (8.00)	.59
Hypertension	34 (85.0)	14 (93.3)	20 (80.0)	.25
Ejection fraction	59.97 ± 6.48	61.13 ± 6.06	59.28 ± 6.75	.39
Cardiovascular intervention	2 (5.00)	1 (6.67)	1 (4.00)	.71
Respiratory failure	27 (67.5)	9 (60.0)	18 (72.0)	.43
Atelectasis	24 (60.0)	7 (46.7)	17 (68.0)	.18
Pleural effusion	25 (62.50)	9 (60.00)	16 (64.00)	.80
Renal failure (RIFLE III)	1 (2.50)	0 (0.00)	1 (4.00)	.43
Diabetes	2 (5.00)	2 (13.33)	0 (0.00)	.06
Medications				
Beta blocker	10 (25.0)	3 (20.0)	7 (28.0)	.57
Anticoagulants	1 (2.50)	0 (0.00)	1 (4.00)	.43
Intraoperative variables				
Surgery type				
Aortic valve replacement or valvuloplasty	5 (12.50)	2 (13.33)	3 (12.00)	.90
Coronary artery bypass grafting	2 (5.00)	1 (6.67)	1 (4.00)	.71
Lowest temperature, °C				
Nasopharyngeal	24.7 ± 0.8	24.7 ± 1.1	24.8 ± 0.7	.71
Rectal	26.4 ± 1.4	25.8 ± 1.4	26.8 ± 1.2	.02
Antegrade cerebral perfusion flow, mL/min/kg	5.48 ± 0.65	5.30 ± 0.31	5.59 ± 0.78	.10
Cardiopulmonary bypass, min	280 ± 58	303 ± 74	267 ± 42	.05
DHCA duration, min	29 ± 6	31 ± 7	28 ± 5	.12
Cross-clamping time, min	169 ± 42	186 ± 51	159 ± 32	.08
Perioperative blood transfusion				
Red blood cells, units	6.56 ± 3.28	7.27 ± 3.97	6.14 ± 2.80	.30
Fresh frozen plasma, units	1.79 ± 1.44	1.92 ± 1.81	1.71 ± 1.20	.67
Platelet, units	1.83 ± 0.45	1.87 ± 0.52	1.80 ± 0.41	.65

POCD occurred in 7 patients on the day after extubation and in another 8 patients on day 7 after surgery. Among all 15 patients with POCD, MMSE score decreased from 26.00 ± 2.85 before surgery to 22.73 ± 4.01 at 1 hour after extubation and then to 20.60 ± 5.00 on day 7. Their nadir MMSE was 19.40 ± 4.60, which was 6 points lower than before surgery. Five patients reached their nadir on the day after extubation, and 10 reached it on day 7 (Table 2). Among patients with POCD, MMSE score did not decrease as a result of surgery and even increased slightly by day 7.

**Table 2:** Scores on the Mini-mental State Examination at different time points.

Time point	Total (n = 40)	POCD (n = 15)	Non-POCD (n = 25)	*P*
Before surgery	24.90 ± 2.91	26.00 ± 2.85	24.24 ± 2.79	.06
After extubation	23.98 ± 3.42	22.73 ± 4.01^a^	24.72 ± 2.85	.08
Day 7 after surgery	24.23 ± 4.29	20.60 ± 5.00^a^	26.40 ± 1.50^b^	<.01
Nadir score after surgery	21.78 ± 4.17	19.40 ± 4.60	23.20 ± 3.20	.01

Patients with or without POCD showed similar clinicodemographic and intraoperative characteristics except that women and patients with lower rectal temperature during circulatory arrest were more likely to suffer POCD (Table 1). Univariate logistic regression identified both variables as risk factors of early POCD: female sex, crude OR 4.89 (95%CI 1.00–23.93); and lower rectal temperature, crude OR 0.53 (95%CI 0.30–0.95). Multivariate regression identified only lower rectal temperature as an independent risk factor (crude OR 0.53, 95%CI 0.30–0.95; adjusted OR 0.53, 95%CI 0.30–0.95), adjusted ORs were calculated after controlling for both gender and rectal temperature.

### 3.1 Peak levels of biomarkers and early POCD

**Figure 2:**
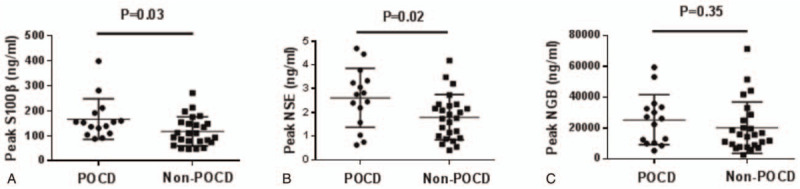
Peak levels of S100β, neuron-specific enolase (NSE), and neuroglobin (NGB) in patients with or without early postoperative cognitive dysfunction (POCD).

Plasma levels of all 3 candidate biomarkers significantly increased after circulatory arrest (Table 3). Levels of NSE and NGB peaked at rewarming to 36°C and returned to baseline by 24 hours after surgery. In contrast, levels of S100β peaked at 6 hours after surgery and remained above baseline even at 24 hours after surgery. Levels of S100β and NSE were significantly higher in POCD patients than in non-POCD patients upon rewarming to 36°C and at least 2 of the time points after CPB. Peak levels of these biomarkers were significantly higher in POCD patients (Fig. 2). Levels of NGB, however, were similar between the 2 groups at all time points, and peak levels were similar between POCD and non-POCD patients.

**Table 3:** Levels of potential plasma biomarkers of early POCD.

				After surgery		
Biomarker	Group	Before surgery	Rewarming to 36°C	1 h	6 h	24 h
S100β (ng/mL)	POCD	0.10 ± 0.04	0.16 ± 0.08^a^	0.17 ± 0.08^b^	0.15 ± 0.08^a^	0.12 ± 0.07
	Non-POCD	0.09 ± 0.04	0.11 ± 0.06^a^	0.12 ± 0.06	0.10 ± 0.04	0.10 ± 0.05
	*P*	.70	.04	.03	.03	.25
NSE (ng/mL)	POCD	1.26 ± 0.99	2.61 ± 1.24^b^	2.42 ± 1.11^b^	2.02 ± 0.86^b^	1.85 ± 1.09^a^
	Non-POCD	0.76 ± 0.69	1.56 ± 0.76^b^	1.52 ± 0.85^b^	1.37 ± 0.78^b^	1.21 ± 0.75^a^
	*P*	.07	<.01	.01	.02	.04
NGB (ng/mL)	POCD	9.90 ± 5.78	25.41 ± 16.16^b^	15.39 ± 7.82^a^	14.50 ± 8.22^b^	8.47 ± 4.19
	Non-POCD	12.43 ± 6.07	20.31 ± 16.61^a^	17.97 ± 10.84^a^	11.07 ± 6.40	10.31 ± 5.70
	*P*	.20	.35	.43	.15	.29

**Figure 3:**
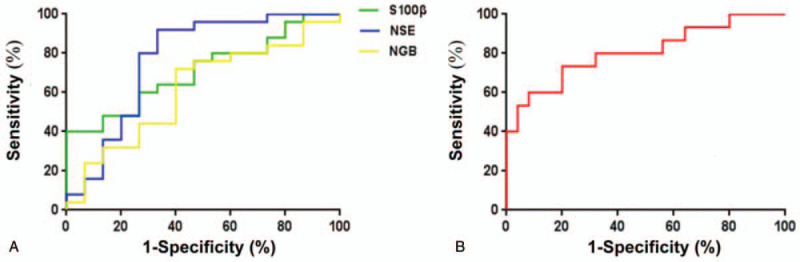
Receiver operating characteristic curves of (A) each potential biomarker on its own or (B) the combination of S100β and neuron-specific enolase (NSE) for predicting early postoperative cognitive dysfunction.

We assessed the ability of S100β and NSE on their own to predict early POCD using receiver operating characteristic curves. For S100β alone, the area under the curve (AUC) was 0.71 (95%CI 0.55–0.87), sensitivity was 48%, and specificity was 87% (Fig. 3A). For NSE alone, AUC was 0.77 (95%CI 0.60–0.94), sensitivity was 92%, and specificity was 67%. The best results were obtained by combining the 2 biomarkers, which gave an AUC of 0.81 (95%CI 0.66–0.96), sensitivity of 73%, and specificity of 80% (Fig. 3B). The AUC for NGB was 0.62 (95%CI 0.43–0.80), confirming its inability to predict POCD.

## 4 Discussion

Using MMSE, which is widely used to screen for cognitive dysfunction^[19]^ and which shows sensitivity of 85% to 97% and specificity of 70% to 90% ,^[20,21]^ we observed POCD in nearly 40% of our sample, consistent with previous observations that early POCD occurs in many patients after total arch replacement surgery under CPB.^[22]^ The present study may aid in early identification of such patients by showing that plasma levels of S100β and NSE, which increased during and after CPB, are associated with early POCD and show potential for predicting it.

While levels of S100β in blood and cerebrospinal fluid are negligible under normal conditions,^[23]^ its levels begin to increase during CPB as early as the rewarming step, they peak at 1 hour after CPB, and then they return to baseline by 24 hours after CPB. This is similar to what has been reported in cardiac surgery with CPB^[24,25]^ and similar to what happens to plasma levels of pro-inflammatory factors such as IL-6 and IL-8.^[26]^ We found that peak levels of S100β were able to predict early POCD with an AUC of 0.71. This is consistent with the idea that S100β can be a specific, sensitive indicator of central nervous system injury.^[27,28]^ The transient increase in S100β in our study, similar to what was observed in a study of coronary artery bypass grafting (CABG),^[29]^ may explain why S100β levels do not appear to be associated with long-term POCD.^[24,25,30]^

Our results suggest that CPB induces changes in glial cells analogous to a systemic inflammatory response, which is consistent with a role for glial cells as “immune cells” in the central nervous system.^[26,27]^ Indeed, our results suggest that CPB induces a neuroinflammatory response that contributes to early neurological complications after cardiac surgery, which is consistent with studies of the inflammatory response after CPB in animal models,^[26]^ comparison of brain injury after cardiac surgery with or without CPB,^[25]^ and clinical analyses of neuroinflammatory factors in brain injury in brain injury patients undergoing CABG^[28,29]^ or valve replacement under CPB.^[30]^

Consistent with this idea, we found that levels of NSE, which is released from damaged neurons during neuroinflammation,^[31,32]^ were also associated with early POCD. NSE levels doubled during CPB as early as rewarming to 36°C and remained high even by 24 hours after CPB. Similarly, studies of patients undergoing CABG or valve replacement with CPB found that NSE began to increase during or immediately after surgery, and the high concentration level lasted until 48 hours after surgery.^[24,33]^ These results suggest that damage induced by CPB may trigger prolonged repair pathways.

In contrast to the neuroinflammation biomarker S100β and the nerve injury biomarker NSE, NGB was not significantly associated with early POCD, even though its levels increased significantly during and after CPB. On one hand, NGB can leak out of damaged neurons^[14]^ and therefore may serve as an index of injury. On the other hand, it may promote the diffusion of oxygen to mitochondria and thereby mitigate hypoxic injury and resist oxidative stress.^[34]^ In this way, for example, it can reduce risk of stroke after cerebral ischemia.^[35]^ Thus, the protective effect of NGB leaked from damaged neurons may render it unreliable as an injury marker for predicting POCD.

Our results should be interpreted with caution given the small sample and the short postoperative follow-up of 5 to 7 days after surgery. In addition, we had to exclude patients with postoperative stroke because we could not assess their neurological function using the MMSE. Future studies with a larger sample and longer follow-up should verify and extend our findings that the combination of S100β and NSE can predict POCD after cardiac surgery. Such work may lead to revision of clinical guidelines for avoiding this potentially debilitating complication.

## Author contributions

**Conceptualization:** Xiaoyan Wang.

**Data curation:** Zilin Wan, Huishun Ye.

**Formal analysis:** Zilin Wan.

**Funding acquisition:** Xiaoyan Wang.

**Investigation:** Zilin Wan, Yaxiong Li, Huishun Ye, Yunfeng Zi.

**Methodology:** Yaxiong Li.

**Project administration:** Yaxiong Li, Xiaoyan Wang.

**Resources:** Yaxiong Li, Huishun Ye, Yunfeng Zi, Xiaoyan Wang.

**Supervision:** Xiaoyan Wang.

**Validation:** Zilin Wan, Yaxiong Li, Guojing Zhang.

**Visualization:** Zilin Wan, Yaxiong Li.

**Writing – original draft:** Zilin Wan.

**Writing – review & editing:** Zilin Wan, Yaxiong Li, Huishun Ye, Yunfeng Zi, Guojing Zhang, Xiaoyan Wang.
